# The Week's Work

**Published:** 1909-11-27

**Authors:** 


### THE WEEK'S WORK.

### THE NORTH STAFFORDSHIRE INFIRMARY.

#### WILL THE GOVERNORS ACT?

THE annual general meeting of the Governors will be held on Thursday, the 25th instant, after we go to press. No circular notice has been sent to each Governor, which is the practice of the best-managed hospitals, and the advertisement of the meeting appearing in the local Press is most meagre and unattractive. Possibly this is the reason why some people seem to think that the great interest aroused by the publication of the report in THE HOSPITAL of October 2 may have subsided, and that few, if any, Governors will be present. We cannot believe that public spirit is so dead in North Staffordshire, for the efficiency of this hospital is necessarily a material factor in securing the health and happiness of some 400,000 people. We await the result of the meeting with interest and confidence.

### OFF THE LINE.

WE have every sympathy with the desire of the younger men engaged in hospital work to incorporate the Hospital Officers' Association under the Companies Acts. We were glad to notice that in the November issue of the *Hospital Gazette* it is there laid down that there are "certain questions which it must be obvious even to the most ambitious-minded member of the Hospital Officers' Association are beyond their power to take action upon." These questions, it states, may be called "the high politics of the hospital world," and they include "the relationship of the voluntary hospitals to the State or the municipality." In view of this wise counsel general surprise is expressed that Mr. Walter Alvey, a Vice-President of the Hospital Officers' Association, at the seventh annual dinner on November 19, in proposing the toast of "The Hospitals," should have made this question of municipal or State control so prominent a feature of his speech. He even went so far as to state, which we deny, that there is a growing feeling that "the work of relieving the sick and injured is emphatically not a work for private charity, but a work belonging either to the municipality or the State." That view is directly opposed to the opinion of those who are best entitled to speak on behalf of the voluntary hospitals of this country. The sentiment, indeed, ill becomes the Secretary of the Charing Cross Hospital, which has received the most generous support in the last few years from the public, and so affords evidence that the principle of the voluntary support of our great hospitals is accepted as a privilege by the majority of Englishmen.

### THE TREND OF LEGISLATION.

SIR WILLIAM COLLINS, the guest of the evening, who replied to Mr. Alvey's toast, though an ardent Radical, is also a keen supporter and worker for hospitals. The hint thrown out by Mr. Alvey for the destruction of the voluntary hospital system did not altogether, if at all, commend itself to Sir William. He merely observed that it was foreseen when local authorities were charged with the duty of attending to the health of school children that great charitable and sociological changes were likely to follow. That is a very useful utterance at the present time, when the great voluntary hospitals of London are considering whether or not they shall agree to undertake work for the County Council and to receive payment for doing it. The wiser heads amongst hospital managers realise the danger pointed out by Sir William Collins, and, whilst expressing their willingness to attend to such school children as may be sent to the hospitals to the extent of their accommodation and present facilities, have declined to accept any payment for this work. It is true that a minority of the hospitals are still considering the question of receiving payment from the County Council; but the more this proposition is examined and understood the less is it likely to commend itself to voluntary hospital managers. The scale of payment—2s. per child, irrespective of the nature of the ailment or the treatment and number of attendances involved—is not one which is calculated to bring pecuniary benefit to the hospital. The acceptance of payment from the County Council by a voluntary hospital will speedily be followed by a request for a voice in its management.

### VOLUNTARY HOSPITAL FINANCE

The only justification for the surrender of the voluntary principle of hospital support in favour of State or rate aid would be the financial breakdown of this system. Before the foundation of the King's Hospital Fund, when the Metropolitan voluntary hospitals had to face every year a deficiency of at least £100,000, which displayed an alarming tendency to increase, some timid minds with limited capacity might have regarded State or rate aid for voluntary hospitals as a practical question. To-day the King's Fund by its work and example has placed the Metropolitan voluntary hospitals upon a sound financial basis. Not only has the King's Fund directly provided an additional voluntary revenue of £150,000 a year, but, in conjunction with the League of Mercy, it has so extended the area of givers that at the end of ten years from its foundation the total revenue of 94 voluntary hospitals in the County of London has increased by upwards of half a million, that is, by nearly 65 per cent. The same remark applies to the voluntary hospitals throughout the United Kingdom. The latest returns show that the revenue of 796 voluntary hospitals in 1906 yielded collectively a surplus over their total expenditure of approximately £352,000. It is remarkable to find a Vice-President of the Hospital Officers' Association departing from the wise principle laid down in its official organ, and displaying such an ignorance of the actual and present financial position and strength of British voluntary hospitals.

### SECRETARIES; AND STATE AID.

There is one further consideration which Mr. Alvey appears to have overlooked. Whatever might be the effect of the substitution of State or rate aid for the voluntary support of British hospitals, one thing is certain. When the time comes, if it should ever arrive—which Heaven forbid!—that by the supineness of the inhabitants of these islands all voluntary hospitals are abolished, not only will it be a bad day for the patients and for all that is best in hospital treatment, its development and economy, but the office of Secretary will certainly disappear with the altered conditions. State hospitals would be under the control of a central body in each great city or county, and each hospital would be administered by a medical superintendent who would take charge of the whole management. We said last week that the voluntary system makes it essential that the Secretary Superintendent should have financial knowledge and the gift of organisation whereby revenue may be reaped in sufficient volume from voluntary gifts. When the State or municipality supplies the funds one treasurer for each city will be able to take charge of the whole of the financial arrangements, and one clerk will no doubt keep the minutes and do the secretarial work of the board or committee responsible for the general administration of all the hospitals in each centre. We mention this point by way of illustration merely, as an indication that Mr. Alvey's forecast, if it is ever fulfilled, must entail the extinction of the hospital secretary. *Quos Deus vult perdere prius dementat.*

### THE PERMANENT HOSPITAL EXHIBITION IN BERLIN.

The educational and practical value of exhibitions are indisputable, and it is remarkable that no attempt has been made in England to organise a permanent exhibition of hospital appliances or of objects of interest to institutional workers. The important question of flooring, which has been so thoroughly discussed in the columns of The Hospital, for example, appears very difficult to decide merely upon written descriptions or block illustrations. Were it possible, however, for any one interested in this question to go to an exhibition where samples and specimens of the various types of flooring actually laid down, or at least demonstrated on a more or less adequate scale could be viewed and inspected, and where full details of the relative values of such flooring could be obtained, the decision would not be so difficult. Such an exhibition, we believe, is permanently established at the Kaiserin Friedrich Haus in Berlin, and is one of the most instructive and interesting of its kind. Here are shown samples and specimens of the most diverse kinds, all of the highest interest to hospital workers, in each case with a short, concise, and clear description attached, while full references are obtainable to the literature. The exhibition, to some extent, is self-supporting. The State, we believe, makes a small grant towards it, realising the value that it bears for hospital workers, but the greater part of the funds for its upkeep it derives from the letting of exhibit spaces to exhibition firms who show their appliances and furniture.

### WHY NOT HERE.

The Berlin Exhibition, which we had the pleasure of inspecting a short while ago, is so excellent that its success should be an encouragement to hospital workers to start something similar on this side. The busy administrator who is on the look-out for improvements can scarcely find the time or opportunity to visit the various institutions and thoroughly inspect them so as to glean from such an inspection hints for the betterment of his own hospital. Such an exhibition as we have outlined would enable him to take into consideration, at the expense of an afternoon's stroll around it, the progress that has been made in the several departments of his work during the past few years. It will give him a far better idea of the relative importance of various improvements than he can possibly derive from written descriptions in textbooks and technical journals. We venture to think, therefore, that it is desirable that such an exhibition should be organised and put upon a permanent basis, somewhat after the fashion of the Tuberculosis Exhibition, which has been so successful. The matter is one which we believe the British Hospitals Association should take into serious consideration as soon as possible.

### "TRUTH" AND THE HOSPITALS.

It is nearly 40 years ago since the first systematic attempt to make Christmas Day in a hospital a day of happiness for the patients, by the personal service of a number of good people who devoted themselves to the patients on that day, was made.

This new departure was not regarded with much favour at the outset, but has now become crystallised into the general practice. This is well, for as we have often shown, a hospital is a place every right feeling man or woman of intelligence should certainly visit on Christmas Day. *Truth* has for 30 years organised an effort which has steadily grown and now provides toys and amusements at Christmas for the whole of the children to be found in metropolitan hospitals, Poor Law infirmaries, workhouses, workhouse schools and receiving homes, orphanages and charities. In this way 31,000 children will be made happy at the approaching Christmas time, and 4,000 ladies have been at work for months making and dressing dolls, making toys and collecting articles calculated to give pleasure to these poor children. So every child found in the wards and establishments in question will be thought of and cared for.

### WAKE UP.

The *Truth* Fund has developed into a great work involving a considerable outlay in time, money and organisation. Indeed, despite all that has been done in the past, we have satisfied ourselves that everything will be done infinitely better this year than ever before. The *Truth* Toy Show at the Albert Hall on December 15 next should excite much more general interest in the lot of these unhappy children than has yet been aroused. We hope it may result in getting many people to take an active interest not merely this Christmas time, but throughout the whole of each year, in the children to be found in Poor Law infirmaries and workhouses especially. Her Royal Highness the Princess of Wales has expressed the deepest interest in the movement, and has contributed liberally to the *Truth* Fund. Mr. R. A. Bennett, the Editor of *Truth*, has shown a whole-hearted devotion in developing this movement to the utmost. We hope he will receive the support and recognition of all classes of residents throughout the metropolis.

### DRUG PRICES AND PRACTITIONERS.

It is very frequently the case still that the practitioner with the largest practice has his prescriptions made up in his own dispensary. The changes in the drug market are therefore of some importance in general practice, and we propose to indicate actual and prospective changes regularly in this column. This year's Budget, by affecting the duty on spirits, has caused tinctures to go up materially in price. Interest in the drug market at the moment centres round opium, as the rapid advance in price is almost without precedent. The price is likely to continue high, owing to the shortage in the Turkish crop during the last two years and the estimated shortage this season. It must be clear that with opium at 20s. per lb. the market in morphine and codeine is in an interesting condition, owing to the keen competition amongst makers at the moment. But for this competition prices would be much higher than they are at present. The makers of codeine are still in agreement, and the recent advances in prices are justifiable. Glycerine is now in such demand for industrial purposes that its price is steadily rising. Cod liver oil and potassium bromide are likely to increase in price. Calomel and santonin give indications of higher prices. We shall be glad to answer questions or co-operate in this matter with any practitioner who is anxious to work his surgery with the maximum of economy.

### A PERSONAL APPEAL.

In the last issue of his interesting *Parish Magazine* (a model little monthly), Canon Horsley, the rector of St. Peter's, Walworth, records the fact that in view of Hospital Sunday he wrote a letter of appeal which was delivered to nearly every tradesman in the parish. In this letter he strongly urged the claims of the Sunday Fund. "The circumstances of our parish," he remarked, "are such that one of our most obvious duties is to make some return to Hospital Sunday Fund in view of the constant applications I have to make to it for in or out treatment in some special hospital, for appliances of all kinds, and for help towards convalescent treatment when our poorer neighbours come to me in their hour of need." This cordial and strenuous support, on lines which we sincerely hope will be followed next year by every parish priest in the kingdom, met with sadly disappointing results, as only subscriptions to the amount of £2 reached Canon Horsley in response to his appeal. This is much to be regretted, for it is likely to discourage others from following the excellent example of the rector of St. Peter's. We trust, however, that the disappointment will not deter friends of the Fund from trying the personal appeal again.

### THE CHURCH AND THE HOSPITALS.

There have been occasions when such appeals, backed by the personal influence of Churchmen of all denominations, have produced better results than they have this year done in Southwark. In Birmingham and in Dublin we believe they have been a factor in considerably increasing the amount of contributions received in aid of the Sunday Fund. There is everything to be said in their favour. The thoughtful clergyman will find arguments in plenty to support them in that most interesting series of sermons which Latimer preached before the Duchess of Suffolk in 1552, and we venture to suggest that in all such appeals stress should be laid on exactly the points which the great preacher used. "Sitis necessitatibus sanctorum communicantes," "Melius dare est quam accipere." What better texts can be found than these to stimulate the eloquence of those who recognise the catholicity of suffering and the need of personal sacrifice? But in order to make the churches and the clergy fully alive to their duties and responsibilities in this matter it seems to us desirable that they should be better represented on hospital boards and councils than they are at present. In Canon Horsley's own district the hospitals have curiously few governors or councilmen who are leaders of religious thought or actively engaged in Church work. That is a matter which demands some attention at the hands of hospital governors, and we earnestly hope that it will be taken into serious consideration in the near future.

